# A smart viral vector for targeted delivery of hydrophobic drugs

**DOI:** 10.1038/s41598-021-86198-y

**Published:** 2021-03-29

**Authors:** Sukanya Ghosh, Manidipa Banerjee

**Affiliations:** Kusuma School of Biological Sciences, Indian Institute of Technology Delhi, Hauz Khas, New Delhi 110016 India

**Keywords:** Cancer, Chemical biology

## Abstract

Targeted delivery of hydrophobic chemotherapeutic drugs to tumor cells remains a fundamental problem in cancer therapy. Effective encapsulation of hydrophobic drugs in nano-vehicles can improve their pharmacokinetics, bioavailability and prevent off-target localization. We have devised a method for easy chemical conjugation and multivalent display of a tumor-homing peptide to virus-like particles of a non-mammalian virus, Flock House Virus (FHV), to engineer it into a smart vehicle for targeted delivery of hydrophobic drugs. This conjugation method provides dual functionalization to the VLPs, first, a 2 kDa PEG spacer arm shields VLPs from immune reactivity, and second, attachment of the tumor homing peptide tLyP-1 chauffeurs the encapsulated hydrophobic drugs to target cells. The fortuitous affinity of the FHV capsid towards hydrophobic molecules, and dependence on Ca^2+^ for maintaining a stable capsid shell, were utilized for incorporation of hydrophobic drugs—doxorubicin and ellipticine—in tLyP-1 conjugated VLPs. The drug release profile from the VLP was observed to be gradual, and strictly endosomal pH dependent. We propose that this accessible platform empowers surface functionalization of VLP with numerous ligands containing terminal cysteines, for generating competent delivery vehicles, antigenic display and other biomedical applications.

## Introduction

Potent therapeutic molecules sometimes fail to make their mark in clinical trials due to their hydrophobic nature, resulting in concomitant difficulty in parenteral administration. Poor aqueous solubility of therapeutic molecules results in lower bioavailability, rapid metabolism, lower retention time in systemic circulation, and adverse clinical effects in patients in consequence. Many effective anticancer drugs are associated with poor water solubility and are therefore difficult to administer^1^. A variety of nanocarriers have been developed to address this limitation^2^, however, endosomal escape remains a rate-limiting step for several of these vehicles^3^. Thus, despite phenomenal advancements in nanoparticle-based delivery system in the recent years, the requirement for smarter packaging and delivery vehicles for hydrophobic drugs persists.

Virus particles are programmed to deliver cargos into host cells in an orchestrated fashion. A large majority of viruses with animal hosts are capable of puncturing endosomal membranes to allow the viral genome access to the cytoplasmic milieu^4^. Virus-like particles (VLPs), which are non-infectious mimics of infectious viruses with a similar quaternary association of capsid proteins, exhibit the same inherent property and can be utilized for deposition of foreign cargo within the cellular cytoplasm^5^. VLPs fabricated from non-enveloped virus capsids are stable, biocompatible and biodegradable, with a uniform structural organization, which makes them good starting points for developing smart-delivery vehicles^6,7^. However, it is essential to address undesirable immune responses elicited by VLPs in order to use them as delivery vehicles^8,9^. The hydrodynamic size range of delivery vehicles is an important consideration for conveyance of cargo to the correct site of action without being cleared en route by the immune system. While VLPs in the smaller size range (< 30 nm diameter) are capable of being internalized^10,11^, larger VLPs are known to undergo phagocytosis, which may result in inflammation, release of cytokines and rapid systemic clearance^12^. Cancerous tissues are heavily infiltrated by immune cells, therefore VLPs used in cancer therapy have to be shielded from being hijacked, before delivery of the payload. As VLPs display a similar geometric pattern of capsid proteins as the parental virus, they often induce strong cellular and humoral immune responses^10^. Previous studies have suggested that coating VLPs with immunologically inert molecules may assist them to bypass immune recognition^13^. Attachment of inert polymeric molecules, like polyethylene glycol (PEG) on the surface of other drug delivery vehicles have been observed to reduce renal clearance and toxicity, and increase in circulation time and bioavailability of the formulation^14–16^. PEGylation further helps in providing an anchor for additional modifications on the surface of VLPs^13^.

Another requirement for developing VLP-based smart nanotherapeutics is to ensure correct cell specific delivery of the cargo, by genetic or chemical modification of the outer capsid surface with targeting moieties. While Rotavirus and Hepatitis B virus (HBV) have intrinsic tropism toward intestinal cells and hepatocytes respectively, and Canine Parvovirus (CPV) binds to transferrin receptors that are naturally overexpressed on cancer cells^17,18^; there are overt safety issues associated with utilizing mammalian viruses, rather than plant or insect viruses, for drug delivery. Modification of non-mammalian viruses with cell specific targeting peptides ensures precise localization since they cannot gain entry into mammalian cells naturally. Targeted drug delivery further reduces systemic toxicity with increased bioavailability and controlled release^19^.

In this work, we attempted to design a viral vector that will simultaneously address concerns related to hydrophobic drug encapsulation, specific delivery, safety and immunogenicity. We utilized virus-like particles of Flock House Virus (FHV), a non-enveloped, icosahedral, RNA insect virus, as a base vehicle for development of a smart delivery vehicle^20^. While native FHV replicates efficiently in cultured *Drosophila* cells^21^; expression of the capsid protein of FHV in insect *Spodoptera frugiperda* (Sf21) cells results in the self-assembly of 180 identical capsid subunits into uniform ~ 30 nm sized VLPs^22^. The size, uniformity and stability of the particles are conducive to drug delivery. For specific localization of the particles, we chemically conjugated a tumor homing peptide “tLyP-1” on the surface exposed loops of the particle. tLyP-1 (CGNKRTR) is a linear, C-terminally truncated version of Lyp-1 (CGNKRTRGC), a circular peptide capable of specifically recognizing tumor cells, lymphatics of certain tumor types, tumor associated macrophages and macrophages in atherosclerotic plaques^23,24^. Binding of Lyp-1 to overexpressed p32/gC1qR on the surface of tumor cells leads to a proteolytic cleavage event, which exposes the C-terminus of the peptide. tLyP-1 subsequently binds to neuropilin receptors NRP-1 and NRP-2, with relatively higher binding affinity for the former^25^. It has been established that tLyP-1 possesses proapoptotic property and is more effective in specific tumor homing^25^.

Although crystal structures depict FHV as inert, impermeable entities^26^, mass spectrometry and encapsulation reactions with the native virus has clearly established that intact particles are capable of dynamic behavior^27^, and can allow entry of alkylating agents in the capsid interior, resulting in the inactivation of the viral genome^28^. An interesting observation was the increased incorporation of hydrophobic alkylating agents compared to charged ones, indicating that the conduit(s) allowing entry of small molecules to the capsid interior are lined with hydrophobic residues^28^, however, existing structural information does not identify any such channels/pathways for entry of foreign molecules^26^. This observation clearly indicates the potential for packaging small molecules inside the dynamic capsids. We therefore attempted to encapsulate two hydrophobic chemotherapeutic drugs—doxorubicin and ellipticine—in tLyP-1 conjugated FHV VLPs and monitored the packaging, targeting and delivery of the drugs. Our data suggests that these engineered viral delivery vehicles are capable of successfully encapsulating and specifically delivering hydrophobic chemotherapeutic drugs to breast cancer cells; and can potentially avoid the immune response associated with viral vectors with repetitive subunit structures.

## Results

### Surface modification of FHV VLPs with a targeting peptide

FHV VLPs are generated from 180 copies of a capsid protein α (43 kDa), which undergoes a post-assembly proteolytic maturation into β (39 kDa) and γ (4 kDa)^20^. The maturation cleavage of all capsid proteins is required for covalent dissociation of γ^27^, which is a membrane penetrating peptide utilized for endosomal disruption during virus entry^29^. The maturation cleavage also occurs in FHV VLPs, allowing them to cause endosomal disruption similar to that executed by the native virus^30^. For optimal attachment of the targeting peptide to the VLP surface, we utilized a modified capsid protein, with a serine to lysine mutation at position 268 (S268K α). K268 is positioned on the outer surface of particles, at the tip of an exposed loop on α, and is ideal for chemical conjugations^31^. S268K α was expressed in Sf21 cells via recombinant baculovirus infection, and self-assembled particles, which were morphologically similar to wildtype FHV VLPs (data not shown), were purified by standard methods.

**Figure 1:**
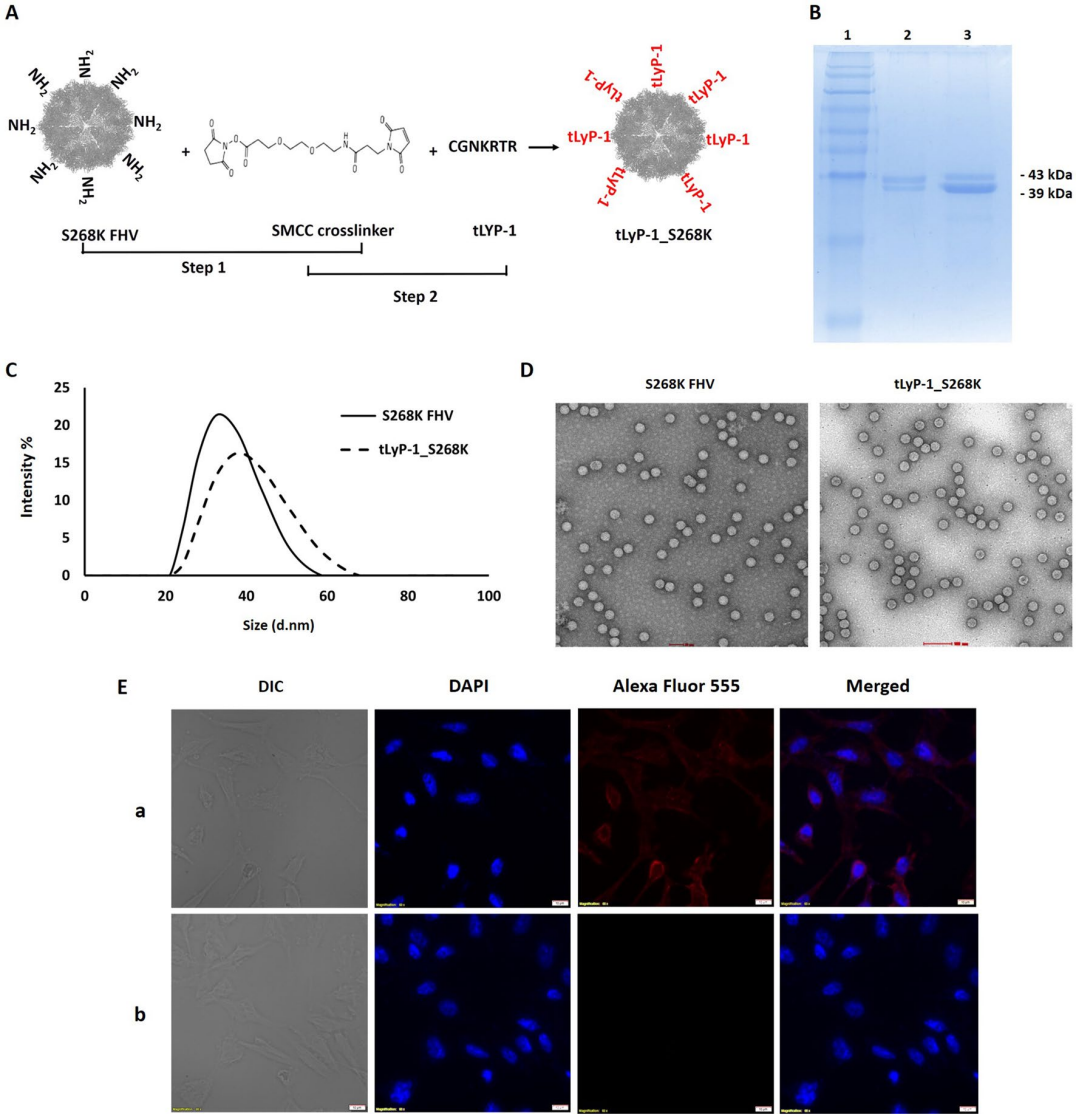
Conjugation of tumor homing peptide tLyP-1 to FHV VLPs (**A**) Schematic representation of tLyP-1 peptide attachment on the surface of S268K FHV VLP (**B**) SDS-PAGE of unconjugated and tLyP-1 conjugated S268K VLPs (**C**) Dynamic light scattering (DLS) of unconjugated and tLyP-1 conjugated S268K VLPs (**D**) Negative stain micrographs of unconjugated and tLyP-1 conjugated S268K VLPs at ×50,000 magnification; (**E**) Confocal microscopy showing binding of tLyP-1 conjugated (panel a) and unconjugated (panel b) S268K VLPs to MDA-MB-231 cells.

The tumor-homing peptide tLyP-1 was attached to the surface of S268K FHV VLPs in two discrete steps. First, a standard NHS (*N*-hydroxysuccinimide) ester-primary amine conjugation was carried out between the K268 residues of VLPs and the NHS ester arm of a heterobifunctional PEGylated SMCC crosslinker, SM(PEG)_2._ The maleimide groups at the free end of the VLP-associated crosslinkers were subsequently allowed to form thioether bonds with the sulfhydryl group of the N-terminal Cysteine residue of tLyP-1 peptides (CGNKRTR). This resulted in the insertion of inert polyethylene glycol (PEG) molecules of 2 kDa between the VLP surface (Fig. 1A) and targeting peptides, which is expected to shield the surface of the capsid from the immune system. The conjugated VLPs (denoted tLyP-1_S268K) were resolved on a 15% SDS-PAGE, along with unconjugated S268K VLPs (Fig. 1B). A very slight increment in molecular weight was observed, since the position of the bands obtained correspond to the monomeric forms of the respective proteins upon denaturation of VLPs. To detect any size difference at particle level, both unconjugated and tLyP-1 conjugated VLPs were subjected to Dynamic Light Scattering (DLS) on a Malvern Zetasizer (Fig. 1C). Hydrodynamic size analysis indicated a clear increase in mean size distribution of conjugated particles, indicating successful attachment of peptides. Negative staining electron microscopy (Fig. 1D) showed that the conjugated particles (right panel) were visually like the unconjugated particles (left panel), indicating that FHV VLPs were morphologically stable following tLyP-1 attachment.

For functional confirmation of tLyP-1 attachment, confocal microscopy was utilized. S268K and tLyP-1_S268K VLPs, at a concentration of 0.1 µM, were added to mammalian breast cancer cell line MDA-MB-231 that has a moderate expression of tLyP-1 specific NRP receptors on its surface^25^. VLPs were allowed to attach to MDA-MB-231 cells for 1 h at 4 °C, followed by washing to remove unattached particles, and labeling of bound particles with a rabbit anti-FHV polyclonal antibody in conjunction with an Alexa Fluor™ 555 conjugated secondary antibody. Fluorescence corresponding Alexa Fluor™ 555 was associated with the cell surface of tLyP-1_S268K treated cells only (Fig. 1E, panel a) indicating that only tLyP-1 conjugated particles are capable of binding to the MDA-MB-231 cells. As expected, the base VLPs (S268K FHV) did not show any ability to bind to the target cells (Fig. 1E, panel b). This indicated that the tLyP-1 conjugated VLPs must express a substantial amount of targeting peptides on their surface, since binding of VLPs to target cells is exclusively mediated by surface attached tLyP-1. Unconjugated FHV VLPs do not bind to mammalian cells in the absence of a targeting ligand (Fig. 1E, panel b).

### Encapsulation of drugs

For encapsulating drugs in the interior of the tLyP-1_S268K VLPs, the capsid structure was partially destabilized. It has been shown earlier that the FHV capsid incorporates Ca^2+^ at the quasi 3-fold axis of symmetry and at subunit interfaces^26^. Removal of Ca^2+^ by EGTA chelation destabilizes the capsid, which is evident from relatively lower melting temperatures, although the shell remains morphologically intact^32^. In order to facilitate the entry of drugs inside the capsid, tLyP-1_S268K VLPs were initially treated with 200 mM EDTA, followed by high molar excess of doxorubicin and ellipticine. After allowing sufficient time for packaging of drugs, tLyP-1_S268K VLPs were dialyzed against a buffer containing CaCl_2,_ for re-sealing of the particles. Particles were extensively dialyzed to remove any excess drugs, and the amounts encapsulated within the capsid shell were quantified using the intrinsic absorbance of doxorubicin and ellipticine. The absorbance of tLyP-1_S268K VLPs encapsulating doxorubicin (denoted Dox_tLyP-1_S268K) and ellipticine (denoted EPT_tLyP-1_S268K) were measured at 495 nm and 310 nm respectively.

**Figure 2:**
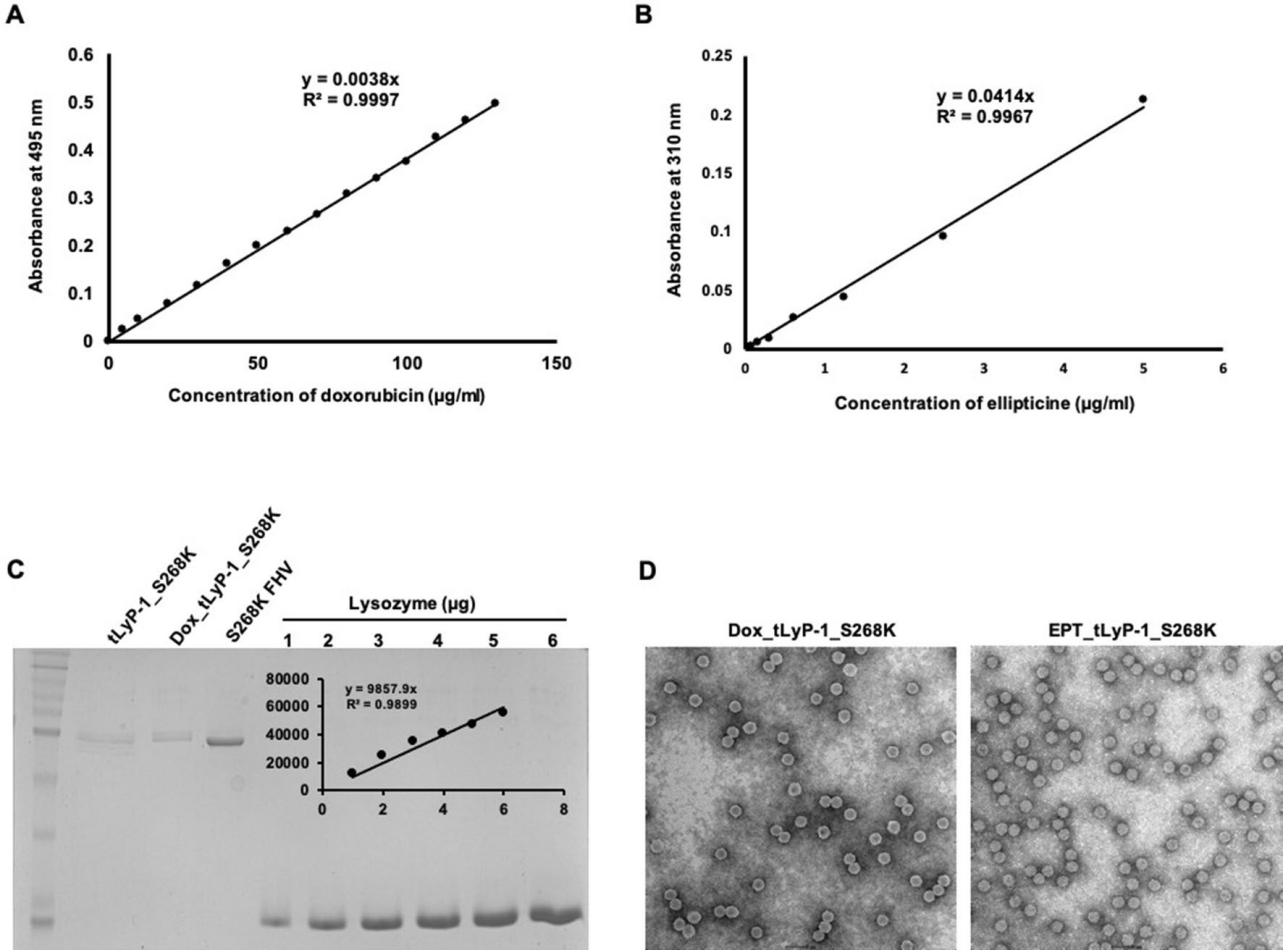
Quantification of doxorubicin and ellipticine encapsulation in VLPs. Standard calibration curve of (**A**) doxorubicin**,** (**B**) ellipticine; (**C**) VLP capsid protein (**D**) Negative stain micrographs of Doxorubicin and Ellipticine encapsulated VLPs, at ×50,000 magnification.

The amount of doxorubicin and ellipticine present in a particle population was quantified from standard curves of the drugs (Fig. 2A,B). tLyP-1_S268K VLPs, in contrast to Dox_tLyP-1_S268K and EPT_tLyP-1_S268K, did not display any quantifiable absorbance at 495 or 310 nm. In order to generate a drug molecule/particle ratio, the capsid protein in the same particle population was quantified by densitometric analysis of denatured VLPs resolved on a 15% SDS-PAGE (Fig. 2C). Our quantification studies suggest encapsulation of ~ 130,000 doxorubicin molecules and ~ 270,000 ellipticine molecules per particle. Negative stain electron microscopy of Dox_tLyP-1_S268K and EPT_tLyP-1_S268K showed that encapsulation of drugs did not cause any obvious structural distortion in VLPs (Fig. 2D). Cryo-electron microscopy of Dox_tLyP-1_S268K and EPT_tLyP-1_S268K also showed that destabilization and resealing of capsids did not affect the overall icosahedral geometry of VLPs (data not shown).

### Drug loaded particles are internalized in MDA-MB-231 cells

**Figure 3:**
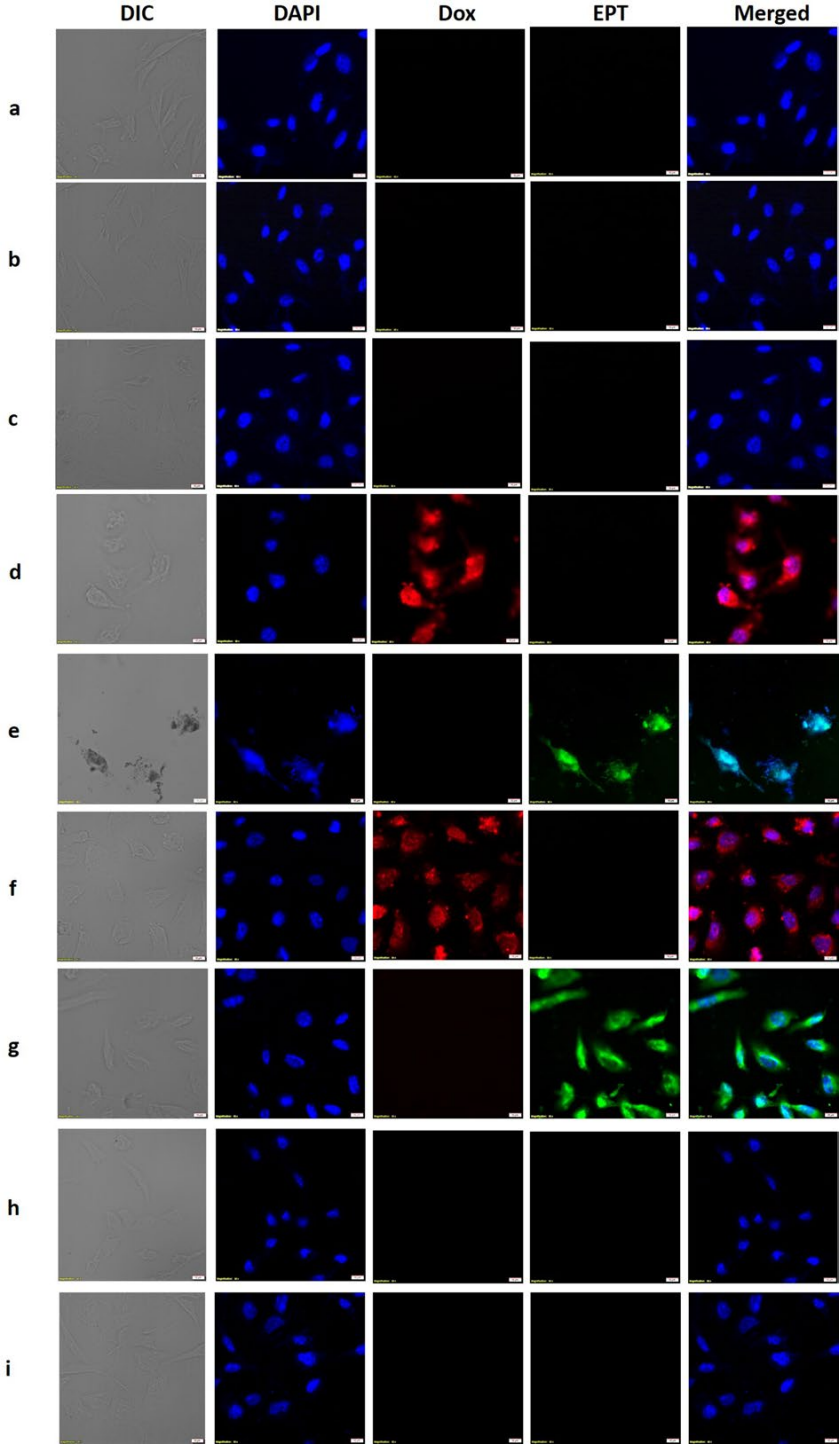
Internalization and distribution of doxorubicin and ellipticine in MDA-MB-231 cells using confocal microscopy. (Panel **a**) corresponds to untreated cells; (panel **b**) to S268K VLP treated cells; (panel **c**) to tLyP-1_S268K treated cells; (panel **d**) to free doxorubicin treated cells; (panel **e**) to ellipticine treated cells; (panel **f**) to Dox_tLyP-1_S268K treated cells and (panel **g**) to EPT_tLyp-1_S268K VLP treated cells. (panel **h,i**) show the effect of ammonium chloride treatment of MDA-MB-231 cells on Dox_tLyP-1_S268K and EPT_tLyp-1_S268K VLP internalization respectively. All images are at ×60 magnification.

The internalization of drugs encapsulated within tLyP-1_S268K VLPs in MDA-MB-231 cells was studied using confocal microscopy (Fig. 3). Cells were first treated with 4 µM doxorubicin or 500 µM ellipticine, and the intrinsic fluorescence of the drugs were exploited to detect their localization (Fig. 3, panels d, e). Both doxorubicin and ellipticine localized to the nucleus at the incubation time of 6 h, as detected by co-staining with DAPI (Fig. 3, panels d, e). Dox_tLyP-1_S268K and EPT_tLyP-1_S268K VLPs, containing the molar equivalent of free drugs, were tested in similar internalization assays. The autofluorescence of doxorubicin and ellipticine encapsulated in particles, were found to co-localize with DAPI fluorescence after 6 h (Fig. 3, panels f, g), indicating the release of drugs from VLP carriers, and their eventual deposition in the nucleus of targeted cells. Similar internalization assays with Dox_tLyP-1_S268K and EPT_ tLyP-1_S268K in the presence of 50 mM ammonium chloride resulted in complete absence of doxorubicin and ellipticine localization in the nucleus of MDA-MB-231 cells (Fig. 3, panels h, i). This indicates that the release of drugs in the cytosol of target cells and consequent localization to the nucleus is dependent upon endosomal acidification, and that the VLPs likely enter cells through the endosomal route.

### In vitro drug release profile from VLPs

**Figure 4:**
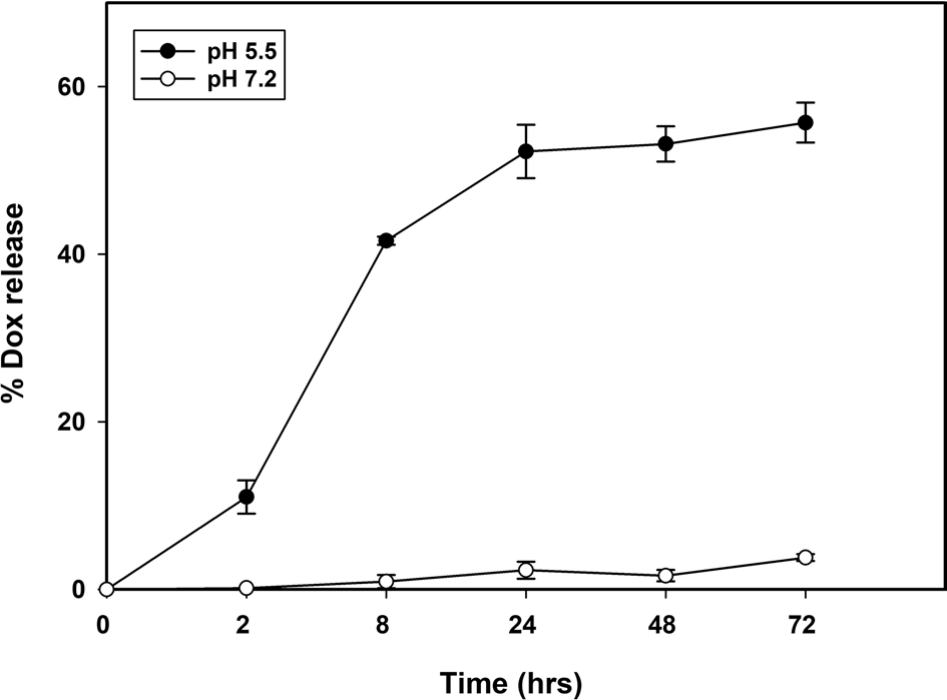
In vitro release profile of doxorubicin from Dox_tLyp-1_S268K VLPs at pH 7.2 and 5.5. Data was collected at 2, 8, 24, 48 and 72 h respectively at 37 °C.

To rule out leaching of drugs from VLPs, and subsequent accumulation in MDA-MB-231 cells by free diffusion, the stability of drug encapsulation in Dox_tLyP-1_S268K VLPs was checked in vitro. No significant release of encapsulated drugs from VLPs was noted after 72 h of incubation at near physiological conditions of temperature and pH (37 °C, pH 7.2) (Fig. 4). In contrast, at a pH corresponding to that of late endosomes (pH 5.5), a gradual release of drug from Dox_tLyP-1_S268K VLPs was noted, with ~ 56% release post 72 h (Fig. 4). The corresponding drug release at physiological pH from the same vehicles was ~ 4%. Since Doxorubicin fluorescence is expected to self-quench at high concentrations; all measurements were carried out at the linear range of fluorescence^33^. It has been shown previously that low endocytic pH is essential for destabilization of FHV capsid, exposure of membrane active regions, and subsequent genome release and virus infectivity^29^. Our modified VLPs behave in a similar manner, with a tight pH control on release of encapsulated material. Taken together with confocal microscopy experiments, this result unequivocally shows that the release of drugs from FHV VLPs occurs along the endosomal route of target cells.

### Dox_tLyP-1_S268K mediated cell cytotoxicity on MDA-MB-231 cells

**Figure 5:**
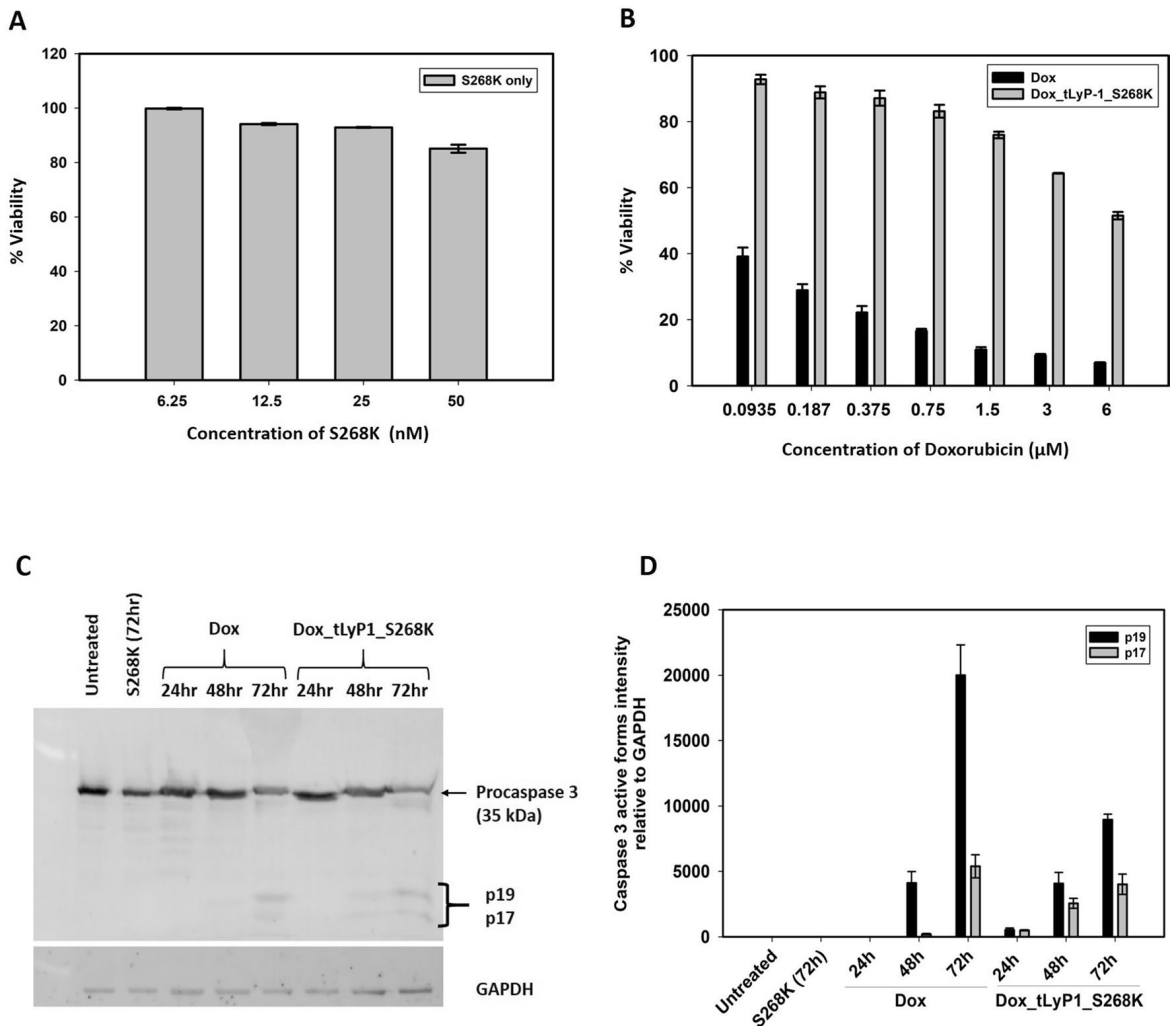
Encapsulated doxorubicin induces apoptosis mediated cell cytotoxicity in MDA-MB-231 cells. Evaluation of cytotoxic effect of (**A**) S268K FHV VLPs and (**B**) free doxorubicin and Dox_tLyp-1_S268K VLPs post 72 h of treatment by MTT assay; (**C**) Immunoblotting showing cleavage of caspase 3 into its active subunits (p17 and p19), and (**D**) the corresponding densitometric analysis. Note: for (**C**,**D**), n = 2 where ‘n’ represents different experimental setups.

In order to assess the cytocompatibility of the base delivery vehicle, different concentrations of S268K FHV VLPs were added to MDA-MB-231 cells for a period of 72 h, followed by a standard MTT assay. The VLPs were found to have significantly high cytocompatibility, as they were non-toxic to MDA-MB-231 cells up to fairly high concentrations. Cell viability was found to be 93% at a concentration of 25 nM of VLPs, which reduced to ~ 85% at double the concentration (Fig. 5A), indicating that the VLP vehicle itself is not likely to have substantial toxic effect on cells, and any cell death observed will be primarily due to the encapsulated drugs. MDA-MB-231 cells were further incubated with increasing concentrations of free doxorubicin and ellipticine, as well as the corresponding molar concentrations of drugs encapsulated in Dox_tLyP-1_S268K and EPT_tLyP-1_S268K VLPs.

With increase in concentration of doxorubicin, viability of MDA-MB-231 cells decreased significantly, as expected. At a doxorubicin concentration of 6 µM, a cell viability of approximately 7% was observed. Interestingly, the cellular viability upon treatment with the corresponding molar equivalent of doxorubicin encapsulated in Dox_tLyP-1_S268K (concentration of VLPs being 1.25 nM) was found to be ~ 50% (Fig. 5B), indicating a tighter control of drug release by the VLPs. It is to be noted that the S268K VLPs themselves did not contribute to cytotoxicity as the VLPs were non-toxic even at five times the concentration (Fig. 5A). The greater cytotoxicity exhibited by free doxorubicin compared to that of the encapsulated population probably indicates that the free drug is capable of nonspecific and passive uptake into cells, in contrast to the drugs encapsulated within VLPs. Further, our in vitro release assay clearly shows that the release of encapsulated drugs from VLP vehicles is gradual, with ~ 56% release after 72 h. PEGylation of the outer VLP surface probably imparts additional stability, resulting in controlled release of doxorubicin from the delivery vehicles. A similar trend of target cell death was observed for free ellipticine and EPT_tLyP-1_S268K VLPs (data not shown).

### Encapsulated drugs orchestrate cell killing by apoptosis

Cancer drugs like doxorubicin are known to cause cell death by triggering apoptosis. In order to determine whether the mechanism of cell death is different when triggered by doxorubicin encapsulating VLPs, an apoptosis assay was performed with 10 µg/ml concentration of free doxorubicin and its molar equivalent encapsulated in Dox_tLyP-1_S268K VLPs. Free drug and VLP vehicles were added to MDA-MB-231 cells, and the proteolytic cleavage of the precursor protein caspase 3 into its active form was monitored by western blotting after 24, 48 and 72 h respectively. While the base VLP vehicle was unable to trigger apoptosis even after 72 h, both free doxorubicin and Dox_tLyP-1_S268K VLPs triggered cleavage of caspase 3 into its active forms as indicated by the appearance of 19 and 17 kDa bands (Fig. 5C). The cleavage of the 33 kDa precursor form of caspase 3 into lower molecular weight bands was evident from the 24 h time point for free doxorubicin and 48 h time point for Dox_tLyP-1_S268K VLPs. (Fig. 5C). Densitometric quantification of the precursor and proteolytically cleaved bands supported this conclusion (Fig. 5D). A slight delay in triggering apoptosis by Dox_tLyP-1_S268K VLPs indicates a more controlled uptake of encapsulated doxorubicin in the cellular milieu.

## Discussion

Almost a hundred years ago, Paul Ehrlich introduced the concept of the magic bullet—a personalized formulation that will address diseases at the molecular level without harming the individual^34^. An embodiment of this concept is an ideal drug delivery system for cancer treatment, which will not harm healthy tissues. The goal to develop a perfect system has seen the convergence of medical, pharmaceutical, bioengineering and material sciences, however, many challenges still remain in this area. Various types of liposome and polymer-based vehicles have been engineered to improved bioavailability of drugs, but surprisingly more than 99% of nanocarriers either accumulate non-specifically or are quickly cleared from the body^35^. Delivery of hydrophobic chemotherapeutic drugs via the intravenous route remains massively challenging as these therapeutic molecules are associated with poor pharmacokinetics and bioavailability^1^. Achieving a fine balance of different molecular characteristics is the key for development of an impactful therapy. In the present study, we have engineered a smart-nanoparticle based biocompatible, non-toxic, stable, targeted drug-delivery system, which can potentially address some of the problems associated with hydrophobic drug encapsulation, targeted delivery to specific locations, immune system reaction and controlled release of encapsulated molecules.

The base vehicle for our smart nanocarrier is the insect nodavirus Flock House Virus (FHV), which does not have any natural tropism for mammalian cells. Attachment of the tumor-homing peptide tLyP-1 to FHV VLPs has resulted in the delivery of encapsulated cancer drugs to the breast cancer cell line MDA-MB-231 and triggered cell death by apoptosis. tLyP-1, chosen as a targeting peptide, is an active CendR peptide that binds to neuropilin receptors (NRP1 and NRP2) that are typically overexpressed in the angiogenic vessels of most malignant tumor cells and in the majority of carcinomas^36^. So far, tLyP-1 has been conjugated to a variety of nano-delivery vehicles based on metal^37,38^, mesoporous silica^39^, PEG-PLA^40^, non-ionic surfactants^41^, liposomes^42,43^ and exosomes^44^. tLyP-1 has also been conjugated with molecular trackers^36,45^, with other peptides^46^, and co-administered with drug-polymer conjugates^47–49^. Conjugation of tLyP-1 to these delivery vehicles has resulted in targeting of tumor imaging agents, as well as therapeutics for treatment of glioblastoma, pulmonary adenocarcinoma and triple negative breast carcinoma^36,40–45,47–49^. tLyP-1 conjugates have been observed to mediate significantly enhanced targeted accumulation of respective therapeutic molecules^36,41,47–49^. To the best of our knowledge, the present study is the first report of tLyP-1 attachment to a virus-based particle by chemical conjugation. Our base particle contains 180 exposed sites for conjugating to tLyP-1. Although we were unable to quantify the exact number of peptides attached per particle, previous studies have shown that ~ 90 molecules of Alexa-488 can be conjugated to S268K FHV VLPs (M. Banerjee, unpublished data). It is likely that steric clashes result in approximately half of potential conjugation sites remaining unattached. Comparative DLS data and confocal-microscopy based delivery assays clearly show that our particles are surface modified with a substantial number of tLyP-1 moieties which facilitate targeting of encapsulated cargo to specific locations. In addition to the tLyP-1 peptide, we have added a PEG coating on the surface of particles, to protect them from detection and clearance by immune cells. It has been shown before that addition of PEG ameliorates the inherent immunogenicity of virus-based delivery vehicles^50^. Other reported beneficial effects of PEG conjugation on delivery vehicles includes increase in stability, enhancement of permeability and retention time, prolonged circulation time, better in-vivo pharmacokinetics and bioavailability and reduction in dose dependent toxicity^42^.

FHV has several properties that make it conducive for biomedical usage. Virus-like particles of FHV can be easily generated in large quantities; the particles are uniform, homogeneous, capable of endosomal membrane penetration and have a stable, adaptable surface which can withstand genetic or chemical modification^20^. Any outer surface modification of FHV hinges on two exposed loop regions on the FHV capsid protein, residues 207–208 and 264–268, which are repeated 180 times on the surface of the particle. These loops have been exploited previously for display of a 181 amino acid long sequence of the anthrax toxin receptor by genetic modification of the capsid protein^31^. Other protein molecules have been chemically attached on to the surface of FHV VLPs by exploiting both covalent and non-covalent interaction chemistry^51^. Our chemical conjugation method is easy, fast and efficient at mild pH and temperature conditions that do not compromise the stability of the VLPs. This method allows multivalent attachment of any protein/peptide with a terminal cysteine residue on the surface of S268K FHV VLPs through a heterobifunctional cross linker containing NHS-ester and maleimide groups. Apart from reducing immune reactivity, it is also possible that the PEG spacer arm may reduce steric hindrance towards attachment of multiple copies of proteins on VLP surface.

The encapsulation of molecules in the capsid interior is a relatively less clear-cut process. Since recombinant production of FHV capsid protein results in the spontaneous assembly of particles in the expression system, incorporation of molecules of choice during in vitro particle assembly is a challenging task. Our laboratory had earlier devised a procedure involving attachment of a long linker to the N-terminal end of the FHV capsid protein to prevent assembly in the expression system. Proteolytic cleavage of the N-terminal linker from purified, monomeric protein triggered assembly and incorporation of small molecules present in the vicinity, within particles^52^. The assembled particles were capable of targeted delivery, in spite of being heterogeneous and non-uniform with a range of sizes. In the current study, we have exploited the dynamicity of fully assembled FHV particles^53^, the preference of the capsid towards hydrophobic molecules^28^ and the role of Ca^2+^ in maintaining capsid integrity^26^, to trigger the incorporation of two hydrophobic drugs inside the icosahedral capsid by partial destabilization of the shell. Electron-microscopy based visualization clearly shows that particles post-encapsulation are stable and icosahedral, indicating that S268K FHV VLPs are competent for undergoing surface modification and enduring partial-destabilization and stabilization transitions. The particles also show a similar pH sensitivity profile corresponding to native FHV, resulting in controlled release of encapsulated material along the endosomal route.

Both doxorubicin and ellipticine are potent inhibitors of topoisomerase II^54,55^. Both drugs have significant side effects, with ellipticine showing high toxicity in clinical trials^56^. Our data shows that while both free and encapsulated drugs can penetrate into the cell nuclei, and trigger cell death by apoptosis; the free drugs are associated with higher cytotoxicity, which is probably due to their relatively smaller size and resultant passive diffusion into cells. A more controlled release of encapsulated drugs within specifically targeted cells is the key to preventing chemotherapy-related side effects, and our study offers a dynamic, smart nano vehicle which can achieve this objective.

## Methods

### Chemicals

Peptides were obtained from GenScript Biotech, USA. SM(PEG)_2_ crosslinker, mammalian cell culture media and reagents were from Thermo Fischer Scientific USA, and doxorubicin hydrochloride (DOX.HCl) and ellipticine from Cayman Chemical. TC-100 insect cell media was from Himedia Laboratories, and BacPAK6 viral DNA (Bsu36I digested) was purchased from Clontech. caspase 3 and GAPDH antibodies were from Cell Signalling Technologies, USA. Carbon coated 200-mesh copper grids were from Electron Microscopy Sciences.

### Cell lines

The insect cell line, *Spodoptera frugiperda* (IPLB-Sf21 or Sf21) were cultured in TC-100 insect cell media supplemented with 10% FBS and 1% penicillin–streptomycin at 27 °C. MDA-MB-231 cells were cultured in L-15 media supplemented with 10% FBS and 1% penicillin–streptomycin at 37 °C.

### Generation of recombinant baculovirus

The cDNA corresponding to FHV capsid protein, cloned in pBacPAK9 transfer vector, was subjected to site-directed mutagenesis using standard protocol (Stratagene) for conversion of Serine268 to Lysine. 1.5 × 10^6^ Sf21 cells in 35-mm tissue culture dishes were co-transfected with 500 ng each of pBacPAK9-S268K FHV capsid and Bsu36I digested linearized BacPAK6 viral DNA, along with 4 μl of Bacfectin transfection reagent in TC-100 serum-free media. After 5 h of incubation, complete growth media was added, followed by a further incubation of 72 h at 27 °C. Transfection supernatant was collected and subjected to plaque purification for isolation of recombinant baculovirus. The initial titre was amplified 3 times in Sf21 cells to generate high titre baculovirus stock for protein expression.

### Expression and purification of S268K FHV VLPs

In order to express S268K FHV capsid protein, 8 × 10^6^ Sf21 cells were infected with high-titre recombinant baculovirus and incubated for 4 days at 27 °C. Cells were lysed with 1% IGEPAL at 4 °C for 20 min followed by centrifugation to remove debris. The supernatant was subjected to ultracentrifugation on a 30% sucrose cushion at 27,3620 g at 11 °C. The pellet was resuspended in a buffer containing 50 mM HEPES pH 7.2 and 5 mM CaCl_2_, and subjected to gradient ultracentrifugation on a 10–40% sucrose gradient at 27,3620 g at 11 °C. The VLP band was dialyzed in 50 mM HEPES pH 7.2, 5 mM CaCl_2_ buffer for removal of sucrose. The concentration of VLPs was calculated using standard methods from optical density (OD) at 260 nm^32^.

### Electron microscopy

5 μl of sample (0.5 mg/ml) was adsorbed onto glow discharged carbon coated 200-mesh copper grids, stained with 2% (wt/vol) uranyl acetate and air-dried. Grids were visualized in a FEI Tecnai G^2^ F20 Transmission Electron Microscope at 200 kV, and images were captured using a 4 k × 4 k CCD camera (FEI Eagle) using TIA software at a magnification of 50,000×.

### Conjugation of tLyP-1 peptides to VLPs

A two-step conjugation reaction was performed to attach tLyP-1 peptides on the surface of S268K FHV VLPs. In the first step, VLPs in 50 mM HEPES, pH 7.2 were incubated with a 50-fold molar excess of SM(PEG)_2_ crosslinker for 2 h at room temperature. After removal of the excess crosslinker by dialysis, SM(PEG)_2_ conjugated VLPs were incubated with 10-fold molar excess of tLyP-1 peptide overnight at 4 °C. tLyP-1 conjugated VLPs (denoted as tLyP-1_S268K) were again subjected to dialysis for removal of unconjugated peptide.

### Drug encapsulation and quantification

Dox base (referred to as doxorubicin) was prepared by dissolving 5 mg of Dox.HCl in 2.4 μl of TEA, followed by addition of 1 ml DMSO and overnight incubation^57^. For encapsulation of drugs, tLyP-1_S268K was dialysed in Tris–EDTA buffer (50 mM Tris at pH 8.0, 200 mM EDTA) at room temperature, followed by incubation with 300 μg of doxorubicin or 200 µg of ellipticine overnight at 4 °C. tLyP-1_S268K encapsulating doxorubicin and ellipticine (denoted as DOX_tLyP-1_S268K and EPT_ tLyP-1_S268K respectively) were further dialysed in Tris CaCl_2_ buffer (50 mM Tris, pH 7.0, 200 mM CaCl_2_) at room temperature, in dark, in order to remove un-encapsulated drug molecules.

The amount of drugs encapsulated in VLPs was quantified using standard calibration curves. Absorbance of doxorubicin and ellipticine were measured at 495 nm and 310 nm respectively using a UV–Vis spectrophotometer. Number of molecules of encapsulated drug per VLP was calculated using free drug standard calibration curves and densitometric analysis of the capsid protein in VLPs resolved on a 15% SDS-PAGE. Protein concentrations were calculated from a standard calibration curve based on densitometric analysis of predetermined concentrations of lysozyme resolved on a 15% SDS-PAGE. All experiments were repeated three times for confirmation of results.

### Size distribution of VLPs

Unconjugated S268K FHV VLPs, and tLyP-1_S268K were subjected to Dynamic Light Scattering (DLS) on a Malvern Zetasizer (Nano ZS90). The average of the mean particle size of three separately generated samples were calculated.

### Confocal laser scanning microscopy (CLSM)

For cellular internalization studies, 8 × 10^4^ MDA-MB-231 cells in L-15 media were seeded on glass slides and incubated overnight. Cells were treated with free drugs, free VLPs, or drug encapsulating VLPs for 6 h at 37 °C. After 3 × washes with DPBS, cells were fixed in 2% paraformaldehyde for 15 min, followed by labeling with 300 nM DAPI for 10 min. After washing in DPBS, cells were mounted and dried. For visualization of VLPs with anti-FHV antibody, a rabbit anti-FHV polyclonal antibody was utilized in conjunction with Alexa Fluor™ 555 labeled goat anti-rabbit antibody. Images were collected in an Olympus confocal laser scanning microscope (Olympus FV1200) at a magnification of 60x.

### In vitro drug release assays

In vitro drug release profiles were determined at both pH 7.2 (cytoplasmic pH) and pH 5.5 (late endosomal pH). 50 µl (0.2 µg/ µl) of Dox_tLyP-1_S268K were dialyzed against PBS (pH 7.2) or sodium acetate buffer (pH 5.5) at 37 °C with gentle stirring**.** At predetermined time points, 200 µl of respective dialysis buffers were sampled out accompanied by replenishment with the same volume of respective fresh buffers. Fluorescence intensities of sampled buffers were measured at λ_ex_ 480 nm and λ_em_ 590 nm (Cary Eclipse Fluorescence Spectrophotometer, Agilent Technologies, Inc.) and the percentage of drug release were calculated using a standard calibration curve. The experiment was performed in triplicates and average values were reported.

### In-vitro cell cytotoxicity assay

For MTT assays, 5 × 10^3^ MDA-MB-231 cells in each well of a 96-well tissue culture plate were treated with different concentrations of S268K FHV VLPs, doxorubicin, ellipticine, Dox_tLyP-1_S268K and EPT_tLyP-1_S268K for 72 h. Media was removed and a 0.5 mg/ml MTT solution was added to the cells, followed by 4 h of incubation in dark at 37 °C. The supernatant was discarded, formazan crystals were dissolved in DMSO and absorbance was measured using a microplate reader (Multiskan GO, Thermo Scientific, U.S.A.) at 570 nm. All experiments were performed in triplicates and repeated three times.

### Immunoblotting assay for apoptosis marker

0.8 × 10^6^ MDA-MB-231 cells, seeded overnight in a 6 cm dish, were treated with equivalent concentrations of S268K FHV VLPs, doxorubicin and Dox_tLyP-1_FHV S268K respectively for 24, 48 and 72 h. Cells were lysed in SDS-RIPA buffer containing a protease inhibitor cocktail and 1 mM DTT. Samples were separated on a 12% SDS-PAGE, followed by western blotting using a caspase 3 primary antibody and a HRP conjugated anti-rabbit secondary antibody. Control blots were carried out with an anti-GAPDH primary antibody. The full-length western blots are shown in Supplementary Figure S1. Images were acquired using a Typhoon FLA 9000 gel imaging scanner and densitometric analysis of active forms of caspase 3 (p19 and p17) were carried out by normalizing the intensity with an endogenous control (GAPDH) using Image J software.

## Conclusions

We have designed a virus-like particle-based nano-vehicle for encapsulation and delivery of hydrophobic drugs to target cells. The vehicles are stable at physiological conditions, and display strict control of targeting and release, suggesting potential inhibition of off-target effects. It is hoped that these particles will prove to be effective in animal models of breast cancer and will show significantly reduced off-target side effects, immunogenicity and toxicity.

## Supplementary Information

Supplementary Information
