# Supplementary material for: A smart viral vector for targeted delivery of hydrophobic drugs
Source: Sci Rep. 2021 Mar 29;11:7030. doi: 10.1038/s41598-021-86198-y (PMC8007742; doi:10.1038/s41598-021-86198-y)

**Supplementary Information**

**A smart viral vector for targeted delivery of hydrophobic drugs**

Sukanya Ghosh, Manidipa Banerjee*

Kusuma School of Biological Sciences, Indian Institute of Technology Delhi, Hauz Khas, New Delhi – 110016, India.

*Correspondence

mbanerjee@bioschool.iitd.ac.in

**Figure S1:** Immunoblotting showing cleavage of caspase 3 into its active subunits p17 and p19 (Original and unprocessed blots)


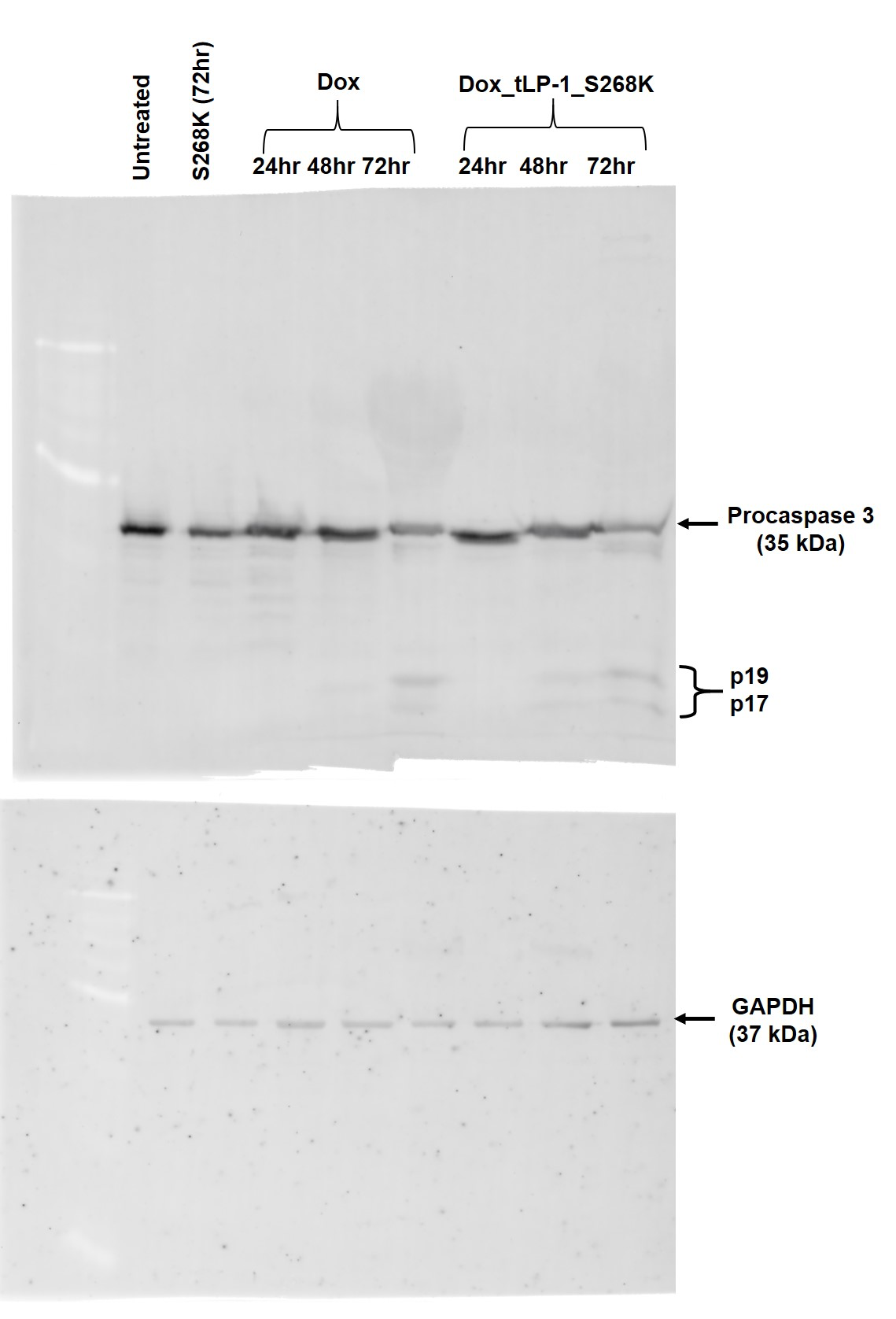

Supplement: Supplementary file 1 — Supplementary Information [file 41598_2021_86198_MOESM1_ESM.docx]
